# *PROCOMIDA*, a Food-Assisted Maternal and Child Health and Nutrition Program, Reduces Child Stunting in Guatemala: A Cluster-Randomized Controlled Intervention Trial

**DOI:** 10.1093/jn/nxy138

**Published:** 2018-08-31

**Authors:** Deanna K Olney, Jef Leroy, Lilia Bliznashka, Marie T Ruel

**Affiliations:** Poverty, Health and Nutrition Division, International Food Policy Research Institute, Washington DC

**Keywords:** children, stunting, Guatemala, food-assisted maternal and child health and nutrition program

## Abstract

**Background:**

Food-assisted maternal and child health and nutrition (FA-MCHN) programs may foster child growth during the first 1000 d (pregnancy and the first 2 y of a child's life), but evidence is scant.

**Objective:**

We evaluated the impact of an FA-MCHN program, *PROCOMIDA*, on linear growth (stunting [length-for-age *z* score (LAZ) < –2] and length-for-age difference [LAD]) among children aged 1–24 mo. *PROCOMIDA* was implemented in Guatemala by Mercy Corps and was available to beneficiaries throughout the first 1000 d.

**Methods:**

We used a longitudinal, cluster-randomized controlled trial with groups varying in family ration sizes [full (FFR), reduced (RFR), and none (NFR)] and individual ration types provided to mothers (pregnancy to 6 mo postpartum) and children (6–24 mo of age) [corn-soy blend (CSB), lipid-based nutrient supplement (LNS), micronutrient powder (MNP)]: *1)* FFR + CSB (*n *= 576); *2)* RFR + CSB (*n *= 575); *3)* NFR + CSB (*n *= 542); *4)* FFR + LNS (*n *= 550); *5)* FFR + MNP (*n *= 587); *6)* control (*n *= 574). Program impacts compared with control, and differential impacts between groups varying family ration size or individual ration type, were assessed through the use of linear mixed-effects models and post hoc simple effect tests (significant if *P *< 0.05).

**Results:**

*PROCOMIDA* significantly reduced stunting at age 1 mo in FFR + CSB, RFR + CSB, and FFR + MNP groups compared with control [5.05, 4.06, and 3.82 percentage points (pp), respectively]. Stunting impact increased by age 24 mo in FFR + CSB and FFR + MNP relative to control (impact = 11.1 and 6.5 pp at age 24 mo, respectively). For CSB recipients, the FFR compared with RFR or NFR significantly reduced stunting (6.47–9.68 pp). CSB reduced stunting significantly more than LNS at age 24 mo (8.12 pp).

**Conclusions:**

FA-MCHN programs can reduce stunting during the first 1000 d, even in relatively energy/food-secure populations. Large family rations with individual rations of CSB or MNP were most effective. The widening of impact as children age highlights the importance of intervening throughout the full first 1000 d.

This trial was registered at clinicaltrials.gov as NCT01072279.

## Introduction

The first 1000 d of a child's life (from pregnancy through 24 mo of age) is a critical window for linear growth and development. Nutrition and health insults during this period can negatively affect children's physical and cognitive development with possible lifelong health and economic consequences (1). Globally, progress has been made in reducing stunting, but some countries still lag behind (2). Guatemala is one such country, where despite the recognition of the problem and the many initiatives put in place to address it, progress to date has been limited (3).

A seminal study begun in the late 1960s demonstrated that providing supplementary food to Guatemalan children in early childhood increased growth, with the largest effects among children who were exposed to the high-energy/protein food supplements in their first 3 y, compared with those exposed when they were 3–7 y of age (4, 5). Furthermore, these benefits were sustained in adolescence and translated into positive human capital and economic outcomes during adulthood (6).

Evidence from supplementary feeding efficacy studies and programs with or without education is mixed but suggests positive effects on linear growth (7). Nutrition education alone has also been shown to increase linear growth, but only in food-secure populations (7). Two efficacy studies have assessed the benefit of providing supplementary food in addition to nutrition education (8, 9) and only 1 showed a significantly greater impact of providing supplementary food in addition to nutrition education on linear growth (9). Some studies also show that the provision of multiple micronutrients to young children improves linear growth, but the size of the effect varies by type of product [lipid-based nutrient supplement (LNS), foodlet, micronutrient powders (MNPs)], maternal and child factors, and study context (9–12).

Interventions during pregnancy, likewise, have had varied efficacy and effectiveness in improving newborn size and early child growth outcomes (13). The provision of supplementary food (14) and multiple micronutrients (15) during pregnancy have been found to reduce the risk of children being small for gestational age. A recent meta-analysis that assessed the provision of multiple micronutrients during pregnancy showed no effect on postnatal growth (16). In Bangladesh, however, multiple micronutrient supplementation during pregnancy reduced stunting at 1 and 3 mo of age (5% and 9%, respectively) but effects were not maintained through 24 mo of age (17).

Given the multifactorial causes of stunting both in pregnancy and after birth, programs designed to address several of these factors are likely to be more effective at reducing stunting and improving linear growth than interventions that focus only on food and/or micronutrient supplementation. The United States Agency for International Development (USAID)/Food for Peace's (FFP's) food-assisted maternal and child health and nutrition (FA-MCHN) program model targeted to women and children during the first 1000 d, Preventing Malnutrition in children under 2 years of age Approach (PM2A), is one such programmatic approach. PM2A was designed to provide women and children in the first 1000 d with a package of interventions aimed to improve household food security and maternal and child diets and health. By simultaneously addressing these underlying and direct causes of undernutrition, PM2A programs aimed specifically to reduce child stunting.

Evidence from an evaluation of USAID/FFP's FA-MCHN program in Haiti demonstrated that targeting the package of interventions to infants and children during the first 1000 d (preventive approach) was indeed more effective at reducing stunting than was targeting children once they had become underweight (recuperative approach) as had been traditionally done (18). However, this program evaluation did not include a control group and thus, the absolute impact of the preventive model on reducing stunting was unknown. Given these promising findings, USAID/FFP commissioned 2 evaluations of their PM2A programs that were to be implemented in Burundi and Guatemala. In addition to being designed to answer the question about the absolute effectiveness of the preventive approach for reducing stunting and its cost-effectiveness, the studies also aimed to answer questions related to how to optimize program impacts and their cost-effectiveness. In Burundi, the evaluation was designed to answer questions about the optimal timing and duration of food supplementation, and in Guatemala the study was designed to answer questions related to the optimal size of the family food ration and the type of micronutrient-fortified individual food ration.

In this article, we present the results from the evaluation of the PM2A program in Guatemala, *PROCOMIDA*. We examine the absolute impact of each of the variations of *PROCOMIDA* on reducing linear growth retardation compared with a control group. In addition, we assess the differential impacts of the size of the family food ration and the type of micronutrient-fortified individual ration. The results from the Burundi program evaluation have been published (19). The results from the cost study will also be published separately.

## Methods

### Program description

*PROCOMIDA* was a USAID/FFP-funded Title II FA-MCHN program implemented in the department of Alta Verapaz in Guatemala and targeted to pregnant women and children during the first 1000 d. Alta Verapaz has a largely indigenous population. Relative to the other 22 departments in Guatemala, it has the highest percentage of households in the lowest wealth quintile (57%), among the lowest levels of education (34% of women and 25% of men have had no education), and the highest levels of illiteracy. Half of children under 5 are stunted in Alta Verapaz (20). However, household hunger is uncommon (<10% of households experience a moderate level of hunger and 0% severe) (21). Overweight and obesity are common in Guatemala, and in Alta Verapaz, more than half of women aged 20–49 are overweight or obese (20).

The key objective of *PROCOMIDA* was to prevent child stunting by delivering sufficient food, promoting the adoption of optimal health, nutrition, and hygiene practices, and improving the provision and utilization of preventive health services. The program aimed to achieve this through 3 main components: *1)* food rations, *2)* a behavior-change communication (BCC) strategy, and *3)* interventions to improve the quality and use of government-funded health services by mothers and children.

Women living in *PROCOMIDA* communities were eligible to enroll in the program when they became pregnant and could participate in the monthly food distributions, BCC sessions, and other program activities from the time of enrollment until their children were 24 mo of age. Over the course of the program, ∼39,000 mother/child pairs participated in *PROCOMIDA* and were served by ∼135 *PROCOMIDA* staff members (unpublished data).

Program beneficiaries received monthly family food rations for the duration of their participation in the program. In addition, beneficiaries received a micronutrient-fortified individual ration which was intended to be consumed daily. The individual ration was provided to women while they were pregnant and up to 6 mo postpartum, at which time the individual ration was targeted to the child until he/she reached 24 mo of age. Receipt of the food rations was conditional on attending the monthly BCC sessions and preventive health services. To receive their monthly rations beneficiaries had to first attend the BCC session, held immediately before the food distribution, and have their health cards checked.

*PROCOMIDA*’s BCC strategy was designed to increase mothers’ knowledge and adoption of optimal health, nutrition, and hygiene practices. Monthly group BCC sessions were led by trained staff and held at health convergence centers (primary health care facilities). Mothers were supposed to be divided into 3 small groups for these sessions (pregnant women, mothers with children 0–5.9 mo of age, and mothers with children 6–24 mo of age); however, this was not always feasible owing to staffing and time constraints. The BCC strategy contained 5 modules, with between 9 and 16 key messages per module. The 5 modules covered the *PROCOMIDA* food commodities, pregnant and breastfeeding mothers (including diet and health of women during pregnancy and early breastfeeding practices), exclusive breastfeeding, feeding and care of children aged 6–24 mo, and feeding (including hygiene practices) and care of sick and/or malnourished children. In addition, cooking demonstrations were held monthly at the homes of leader mothers and focused on the creation of diverse nutritious recipes, most of which utilized the foods provided by the program.

The preventive health component consisted of additional training provided to health service providers to improve quality of service delivery, and the promotion of use of preventive health services by program participants (pre- and postnatal check-ups for women during pregnancy and monthly growth monitoring and promotion for children up to 24 mo of age). *PROCOMIDA* also encouraged utilization of other available preventive health services (e.g., vaccination, deworming) and care-seeking in the case of illness during pregnancy or childhood.

### Study design, participants, and sample size

#### Study design

*PROCOMIDA* was evaluated via a longitudinal, cluster-randomized controlled trial with repeated measures. For the randomization, 120 (out of 215) eligible health convergence centers were stratified by size and randomly assigned to 1 of 6 study groups (20 health convergence centers per study group) that varied the size of the family ration (either full, reduced, or none) and the type of individual ration [corn-soy blend (CSB), LNS, or MNP]. These 6 groups were: full family ration + CSB (FFR + CSB); reduced family ration + CSB (RFR + CSB); no family ration + CSB (NFR + CSB); full family ration + LNS (FFR + LNS); full family ration + MNP (FFR + MNP); and control, which received no *PROCOMIDA* interventions (**Table 1**). Health convergence centers were stratified by size because larger health convergence centers generally served more communities and a larger population. For the larger health convergence centers, *PROCOMIDA* had twice the number of field staff.

**TABLE 1:** Interventions provided by the *PROCOMIDA* program in Guatemala to each study group^1^

	Study groups					
Program component	FFR + CSB	RFR + CSB	NFR + CSB	FFR + LNS	FFR + MNP	Control
Food ration						—
Family ration (rice, beans, oil)	Yes	Reduced	—	Yes	Yes	—
Individual ration	Yes	Yes	Yes	Yes	Yes	—
CSB	Yes	Yes	Yes	—	—	—
LNS	—	—	—	Yes	—	—
MNP	—	—	—	—	Yes	—
BCC	Yes	Yes	Yes	Yes	Yes	—
Required health visits	Yes	Yes	Yes	Yes	Yes	—

^1^Households in the control group had access to the standard health services. BCC, behavior-change communication; CSB, corn-soy blend; FFR, full family ration; LNS, lipid-based nutrient supplement; MNP, micronutrient powder; NFR, no family ration; RFR, reduced family ration.

#### Study participants

All pregnant women who resided in the communities served by the 100 *PROCOMIDA* health convergence centers were eligible to participate in the program and to enroll in the study. Women were invited to enroll in the study when they were identified as pregnant (gestational age 3–7 mo). If they agreed to participate in the study, consent was taken and the enrollment interview conducted. Follow-up interviews were conducted when the child reached 1, 4, 6, 9, 12, 18, and 24 mo of age. Pregnant women living in the communities served by the 20 “control” health convergence centers (not serviced by the *PROCOMIDA* program) were not eligible to participate in the program but were invited to enroll in the evaluation study's control group and had access to the standard government health services. Because our objective was to estimate the intent-to-treat effect, inclusion in the survey was based on program eligibility and not on actual program participation. The study enrollment was thus done separately from program enrollment, and generally preceded it. Pregnant women were invited to enroll in the study until the target sample size was reached. In households with >1 eligible pregnant woman, pregnant women were listed in alphabetic order per their first name, and the first pregnant woman on the list was selected as the “index mother” for the evaluation. If the “index mother” had twins, 1 child was randomly selected as the “index child” by ranking the children's first names alphabetically. The enrollment survey was conducted between August 2011 and December 2012, and the 24 mo survey was conducted between September 2013 and May 2015.

This study was designed to answer 3 primary research questions. The first addressed the absolute impact of the program. To answer this question, each of the program variations was compared with control. FFR + CSB was the primary program package. This package was provided to program beneficiaries served by the 20 health convergence centers allocated to the FFR + CSB group as well as the 95 *PROCOMIDA* health convergence centers not included in the study. Thus, impacts in the FFR + CSB group can be considered as the impacts of the primary *PROCOMIDA* program. The second question addressed the differential impacts of the different family ration sizes. To answer this question, we compared the FFR + CSB group with the RFR + CSB and NFR + CSB groups. The last question sought to determine the most effective micronutrient-fortified individual ration for reducing stunting in this context. To answer this question, we compared the groups that received the FFR + CSB with the groups that received FFR + MNP and FFR + LNS as well as the FFR + MNP with the FFR + LNS group.

Beneficiaries in the FFR groups received 6 kg of rice, 4 kg of beans, and 1.85 kg of vegetable oil every month, while those in the RFR group received about half as much of each food commodity (**Supplemental Table 1**). These amounts were standard across all beneficiaries, regardless of household size or composition. The monthly individual ration consisted of 4 kg of CSB (4 kg), LNS (30 sachets of 20 g each for pregnant and lactating mothers or 60 sachets—meant to be provided 2 times/d—of 10 g each for children aged 6–24 mo), or 60 sachets of MNP. The nutrient compositions of the LNS and MNP were formulated separately for pregnant and lactating women and for children 6–24 mo of age. For each group, the micronutrient compositions of the LNS and MNP were identical but differed in caloric and macronutrient content (**Table 2**). To make the micronutrient content of the MNP the same as that for LNS, macrominerals such as potassium, calcium, magnesium, and phosphorus, which are not typically included in MNPs, had to be added as well as higher concentrations of some micronutrients (e.g., iron and zinc). Both factors changed the organoleptic properties of the MNP supplements. For this reason, we conducted an acceptability trial before the study to test acceptability of the supplements. We found that the MNP supplements were better accepted by pregnant and lactating women and by children when split into 2 doses than when including the whole dose in 1 sitting (22).

**TABLE 2:** Composition of LNS and MNP provided as the individual ration to children (6–24 mo of age) and mothers (pregnant and up to 6 mo postpartum) in 2 of the study groups in the *PROCOMIDA* program in Guatemala^1^

	LNS		MNP	
	Child	Mother	Child	Mother
Daily dose	20 g (2 × 10-g sachets)	20 g (1 sachet)	4 g (2 × 2-g sachets)	4 g (2 × 2-g sachets)
Energy, kcal	118	118	—	—
Proteins, g	2.6	2.6	—	—
Fat, g	9.6	10.0	—	—
Linoleic acid, g	4.5	4.6	—	—
α-Linolenic acid, g	0.6	0.6	—	—
Vitamin A, μg	400	800	400	800
Vitamin C, mg	30	100	30	100
Vitamin D, mg	5	10	5	10
Vitamin E, mg	6	20	6	20
Vitamin K, mg	30	45	30	45
Thiamine (B-1), mg	0.5	2.8	0.5	2.8
Riboflavin (B-2), mg	0.5	2.8	0.5	2.8
Niacin, mg	6	36	6	36
Pantothenic acid (B-5), mg	2	7	2	7
Vitamin B-6, mg	0.5	3.8	0.5	3.8
Folic acid, μg	150	400	150	400
Vitamin B-12, μg	0.9	5.2	0.9	5.2
Iron,^2^ mg	9	20	9	20
Zinc,^3^ mg	8	30	8	30
Copper, mg	0.3	4.0	0.3	4.0
Selenium, μg	20	130	20	130
Iodine, μg	90	250	90	250
Calcium, mg	280	280	280	280
Magnesium, mg	40	65	40	65
Manganese, mg	1.2	2.6	1.2	2.6
Phosphorus, mg	190	190	190	190
Potassium, mg	200	200	200	200

^1^LNS, lipid-based nutrient supplement; MNP, micronutrient powder.

^2^The form of iron in the LNS and MNP was ferrous sulphate and ferrous orthophosphate, respectively.

^3^The form of zinc in the LNS and MNP was zinc sulphate and zinc gluconate, respectively.

Information about the study was provided to potential participants by trained fieldworkers. Informed consent for participation was obtained from either the household head or the index mother. The protocol was approved by the Institutional Review Board of the International Food Policy Research Institute (IFPRI), by Zugueme, an independent ethics committee in Guatemala, and by the National Ethics Committee in Guatemala. The trial was registered at clinicaltrials.gov as NCT01072279.

#### Sample size

The sample size for the study was calculated to detect changes in length-for-age *z* score (LAZ) via the following parameters: a type 1 error of 0.05, power of 0.90, and an intracluster correlation of 0.007. The estimated effect sizes used for sample size calculations differed by the size of the family ration, and whether the comparison was with control, or another treatment group. The largest impact was expected in the groups that received the FFR with CSB, LNS, or MNP. The effect size used (0.339 LAZ) was based on the effect of a preventive compared with a recuperative program in Haiti that included a large family ration (23) and resulted in a sample size of 270/group. Smaller effects were expected in the RFR (0.325 LAZ) and NFR (0.310 LAZ) groups and resulted in sample size estimates of 295 and 329, respectively. Even smaller differences were expected for comparisons between groups. However, given the study budget, we estimated the feasible sample size to be 600 children/group and calculated a detectable difference of 0.263 *z* scores for between-group comparisons. Pregnant women were recruited with the objective of achieving a total of 600 mother-child pairs/treatment group, with adjustments made over the enrollment period to account for attrition. The total sample sizes per group at enrollment ranged from 739 to 794.

### Data collection and measures

Data were collected through the use of Surveybe (a computer-assisted personal interview software produced by Economic Development Initiatives) on portable computers. Surveys were programmed in Spanish and questions translated to Q'eqchi’ directly during the interviews. Extensive training on basic computer skills, the use of Surveybe, and the content of the survey was provided to enumerators over 3 wk with the use of a variety of methods including lectures, role-play, and discussions. Translations of the questions into Q'eqchi’ were practiced and agreed upon during the training. Anthropometrists were trained and standardized (24) in 1 wk. Periodic refresher training and standardization exercises were conducted throughout the study period to ensure high-quality data collection.

#### Household surveys

We used household questionnaires to collect data on household characteristics (e.g., composition, education, housing, food security), maternal characteristics (e.g., age, education level, literacy, height, and weight), and child characteristics (e.g., age and sex). In regard to maternal care for herself and her child, we evaluated adoption of key practices promoted by the program such as use of the individual rations, infant and young child feeding (IYCF) and hygiene practices, and use of preventive and curative health services.

Household food security was assessed at enrollment, 12, and 24 mo with the use of the Food and Nutrition Technical Assistance III (FANTA) Household Hunger Scale (HHS) (25). Household (interior and exterior), mother, and child hygiene were assessed at the enrollment, 12, and 24 mo survey time points through the use of spot checks. Assessment of interior household cleanliness considered whether floors were swept, absence/presence of dirty clothes and animal feces, and if water was covered. Exterior household cleanliness considered whether the yard needed to be cleaned and absence/presence of feces and garbage. For mothers and children, the cleanliness of their hands, faces, and clothes was assessed. Houses, mothers, and children were categorized as “clean” if they were observed to be “clean” in all categories. Use of preventive health services was based on maternal report or recorded from the child health card (if available). For mothers, use of preventive services (pre-, peri-, and postnatal) was assessed at the enrollment, 1, 4, and 6 mo surveys. For children, preventive health service use was assessed at all postnatal surveys and centered on attendance at monthly growth monitoring visits.

Use of CSB was assessed for women at 4 and 6 mo postpartum and for children when they were 6, 9, 12, 18, and 24 mo of age. At each survey time point we asked about intake in the previous 24 h and number of days of intake in the past week (7 d). Intake of LNS and MNP for children at 6, 9, 12, 18, and 24 mo was assessed in the same way as described for CSB. Children would begin receiving the individual ration at the monthly distribution after turning 6 mo of age. Surveys were conducted as close to the child turning 6 mo of age as possible, and thus, the 6 mo survey in most cases would precede the receipt of the child formulations of the MNP and LNS. Use of LNS and MNP among mothers was assessed somewhat differently. At enrollment and 1 mo, women were asked if they had used LNS or MNP during pregnancy. At the 1, 4, and 6 mo time points, mothers were asked if they had used LNS or MNP in the past 24 h. All rations were expected to be used daily by mothers up to 6 mo postpartum and by children 6–24 mo of age. WHO indicators for IYCF practices were constructed according to established guidelines based on maternal recall (26).

#### Clinical assessments

Children's length and weight were measured at all surveys starting at 1 mo. Recumbent length was measured twice and a third length measurement was taken if the difference between the first 2 measurements exceeded 6 mm. The absolute differences between measurements were calculated, and the mean of the 2 measurements with the smallest difference was used to calculate child's length. LAZ was calculated based on the 2006 WHO Growth Standards (27). Stunting was defined as LAZ <–2 SDs. The statistical validity of the use of LAZ in longitudinal analyses has been questioned (28). We therefore also assessed program impact on length-for-age difference (LAD), which is the difference between the child's length and the median length for the child's age and sex from the WHO 2006 International Growth Standard (28).

### Statistical analysis

Statistical analyses were conducted with STATA 14 (StataCorp LP). Household and mother demographic characteristics, and housing quality at enrollment were compared between treatment groups and control.

Program impacts were based on intent-to-treat and were estimated through the use of linear mixed models with child as a random effect (i.e., random intercept by child) and child gender, maternal age and height, and other covariates as fixed effects. All specifications controlled for household, household head, and maternal characteristics at enrollment: a wealth index, constructed through the use of principal components analysis (29), whether the household head and mother had any formal education and spoke Spanish, and the household's dependency ratio. SEs were adjusted for clustering at the health convergence center level, the unit of randomization. Post hoc simple effects tests estimated effects at individual time points. One-sided tests were used to assess program impacts on growth comparing each treatment group with control. Two-sided tests were used for comparisons between treatment groups. Use of CSB, MNP, and LNS were also assessed via linear mixed models and post hoc simple effects tests to estimate differences in supplement use at individual time points. Differences were considered statistically significant if *P* < 0.05.

The primary analyses included children with complete data at all time points and who were measured within 1 mo of each age time point. This age cutoff was used to balance loss of sample size and any potential bias due to including children that were measured well outside an individual age time point given the established relation between age and linear growth between 1 and 24 mo of age. Robustness checks for program impacts were conducted by re-running the linear mixed-effects model allowing the model to treat growth variables as missing if the measurement was taken >1 mo after the age time point.

## Results

### Attrition

**FIGURE 1:**
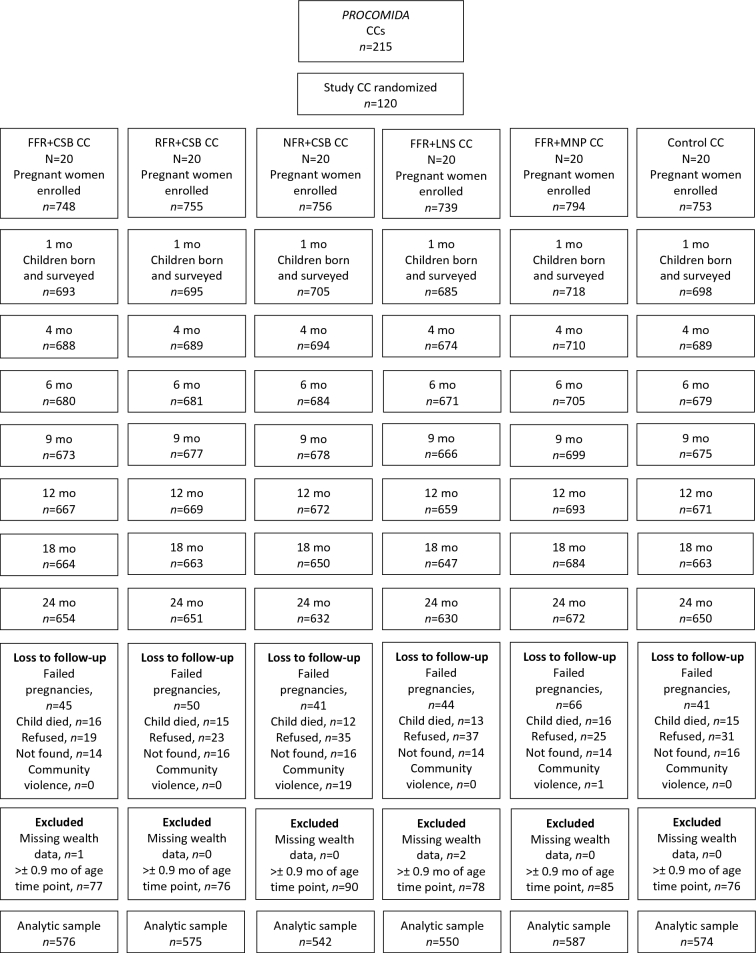
Study flow diagram. CC, convergence center; CSB, corn-soy blend; FFR, full family ration; LNS, lipid-based nutrient supplement; MNP, micronutrient powder; NFR, no family ration; RFR, reduced family ration.

At enrollment, between 739 and 794 pregnant women were interviewed per group (**Figure 1**). At 24 mo, between 84% and 87% of children had data at all time points. In addition to children who were lost to follow-up, between 6% and 12% were excluded owing to having growth measurements >1 mo after the age time point or having missing wealth data, resulting in between 542 and 587 children/treatment group for the primary analytic sample. The proportion of children excluded from the final analytic sample did not differ between groups.

Households excluded from the analytic sample were on average larger, had older household heads, and were more likely to have a household head or mother that had not had any education other than preschool compared with those that were included in the sample (**Supplemental Table 2**). Within treatment groups, there were very few differences in household or maternal characteristics between those included or not, and these were largely confined to the group that did not receive a household ration (NFR + CSB). In that group, excluded households had older household heads and less educated household heads and mothers. The only other differences within treatment groups were limited to larger household size in the FFR + CSB group and to less educated mothers in the FFR + MNP group for excluded compared with included households (data not shown).

### Sample characteristics

The average household size in the study sample was between 6.1 and 6.4 (**Table 3**). Nearly all households owned their home, and most had dirt floors and wood walls. Less than 10% of households were moderately or severely food insecure (i.e., had moderate or severe household hunger). Household heads were nearly all male and were a little less than 40 y old on average and a little less than half had not had any formal education. Mothers were ∼25 y of age and nearly all were married or living with their partner. About one-third of mothers had no formal education and about one-third could speak Spanish. The only significant differences between treatment and control groups were in the proportion of households that had a dirt floor and the proportion of household heads that had not had any formal education. Households in the NFR + CSB group and FFR + LNS group were less likely to have a dirt floor compared with the control group. Household heads in the FFR + MNP group were more likely to have not had any formal education compared with the control group.

**TABLE 3:** Enrollment characteristics of Guatemalan households and mothers included in the analytic sample by study group^1^

	FFR + CSB	RFR + CSB	NFR + CSB	FFR + LNS	FFR + MNP	Control
	(*n* = 576)	(*n* = 575)	(*n* = 542)	(*n* = 550)	(*n* = 587)	(*n *= 574)
Household and housing characteristics						
Size	6.2 ± 3.0	6.2 ± 3.0	6.2 ± 3.0	6.4 ± 3.0	6.1 ± 2.9	6.1 ± 2.9
Owns home	98.3	97.4	98.5	96.9	98.1	97.0
Has dirt floor	89.4	81.0	76.9*	77.8*	80.2	87.1
Has wood walls	68.4	67.7	63.7	68.2	75.8	75.7
Moderate/severe hunger	8.2	10.3	8.5	4.5*	6.0*	10.3
Household heads’ characteristics						
Age, y	39.5 ± 14.2	38.9 ± 13.3	39.8 ± 14.0	40.4 ± 14.1	38.7 ± 13.0	38.9 ± 14.2
Male	93.9	94.8	95.6	93.8	91.1	95.1
Is indigenous	99.5	99.5	99.6	99.8	99.8	99.7
Speaks Spanish	41.1	40.0	46.1	51.8	43.8	43.7
Has not had any education or only preschool	49.8	47.7	41.0	37.8	50.8*	43.9
Mothers’ characteristics						
Age, y	24.6 ± 6.6	24.8 ± 6.4	24.5 ± 6.3	24.8 ± 6.2	24.9 ± 6.6	25.2 ± 6.5
Married/relationship and living with husband/partner	96.4	96.9	95.6	94.0	97.4	96.0
Is indigenous	99.5	99.1	99.6	99.1	99.8	99.5
Speaks Spanish	28.1	33.0	32.5	40.0	30.7	28.2
Has not had any education or only preschool	36.1	37.6	30.4	29.1	34.2	32.2
Height, cm	146.8 ± 4.6	147.2 ± 4.6	147.0 ± 4.6	146.4 ± 4.6	146.9 ± 4.6	146.7 ± 4.8

^1^All values are means ± SDs or percentages unless otherwise indicated. *Different from the control group, *P* < 0.05. CSB, corn-soy blend; FFR, full family ration; LNS, lipid-based nutrient supplement; MNP, micronutrient powder; NFR, no family ration; RFR, reduced family ration.

**FIGURE 2:**
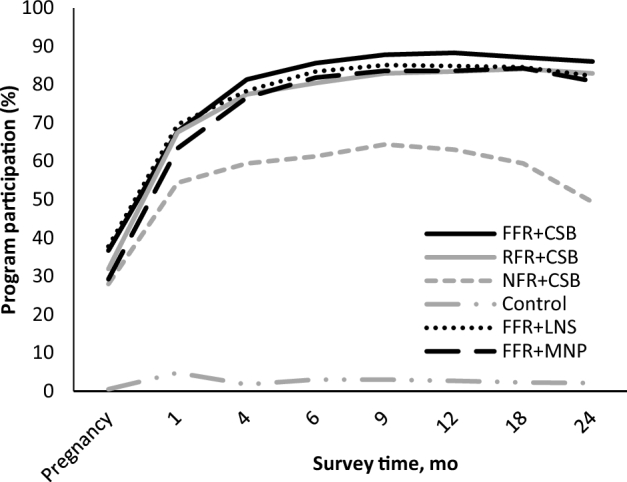
Proportion of eligible beneficiaries participating in *PROCOMIDA* at each time point by study group. Participation was assessed by self-report of participation in the *PROCOMIDA* program. Enrollment into the survey was done during pregnancy and when the child was 1, 4, 6, 9, 12, 18, and 24 mo of age. Among those currently enrolled in *PROCOMIDA*, participation in the monthly behavior change communication and food distributions was >95% at all time points for all study groups. CSB, corn-soy blend; FFR, full family ration; LNS, lipid-based nutrient supplement; MNP, micronutrient powder; NFR, no family ration; RFR, reduced family ration.

At study enrollment (during pregnancy), self-reported program participation was relatively low across the treatment groups (28–38%) (**Figure 2**). However, study enrollment often preceded program enrollment and thus, participation during pregnancy was likely higher than that reported during the enrollment survey. At the 1-mo survey, between 54% and 70% of eligible beneficiaries were participating in *PROCOMIDA*. Program participation increased further by the 4 mo survey and then remained relatively stable between the 4- and 24-mo surveys (at high participation rates, between 77% and 88%), except for the NFR + CSB group, which remained significantly lower throughout the study, between 28% at enrollment and 64% at 12 mo, and declined between the 18- and 24-mo surveys (63% to 49%). No more than 5% of survey participants in the control group reported participating in *PROCOMIDA* at any survey time point, indicating little to no contamination of the control group.

Among those enrolled in *PROCOMIDA*, >95% of participants in all study groups reportedly participated in the monthly BCC sessions and food distributions at all time points and this did not vary by treatment group (unpublished data). Use of the individual rations, however, varied by the size of the family ration and by the type of individual ration. The largest family ration size resulted in the greatest use of CSB by mothers and children. Mothers in the FFR + CSB group compared with those in the NFR + CSB group were significantly more likely to have had CSB in the past 24 h and to have used it more frequently in the past week at both the 4- and 6-mo surveys. A similar pattern was seen for CSB use among children at the 6-, 9-, 12-, 18-, and 24-mo time points. There were no significant differences in CSB use by mothers or children between the FFR + CSB and RFR + CSB groups (**Supplemental Table 3**).

The type of individual ration also influenced reported use of the individual rations. A significantly higher proportion of mothers in the LNS and MNP groups compared with those in the CSB group used their assigned individual ration in the past 24 h and used them more frequently in the past week. A similar pattern was seen among children, apart from the 6-mo time point when the recipient of the individual ration changed from mother to child. At the 9-, 12-, 18-, and 24-mo surveys, a significantly higher proportion of children in the LNS and MNP groups had used their assigned individual ration in the past 24 h (51–66%) compared with those in the CSB group (38–44%). In addition, children in the LNS and MNP groups had used their assigned individual rations more frequently in the past week than those in the CSB group (4 times or 3–4 times, respectively, compared with ∼2 times per wk).

There were no differences in reported use of LNS and MNP among mothers. Children in the LNS group, compared with the MNP group, were more likely to have used their assigned supplement at 9 mo and to have used it more frequently at 9- and 12-mo. However, there were no reported differences in use of LNS and MNP at the 18- or 24-mo time points (**Supplemental Table 4**).

### Program impact

**FIGURE 3:**
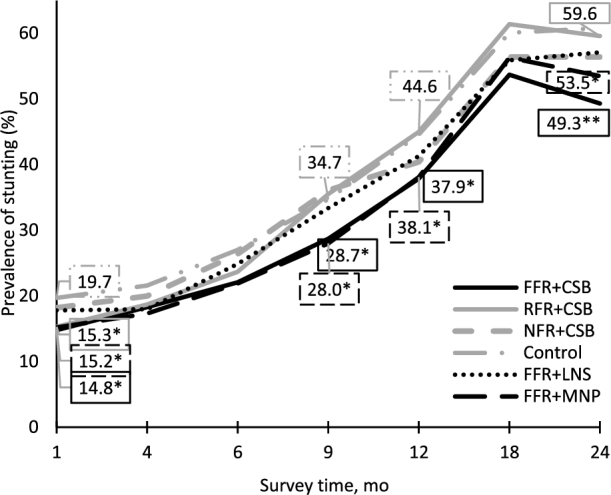
Estimated marginal mean prevalence of stunting (length-for-age *z* score <−2) among children in treatment compared with control groups at 1, 4, 9, 12, 18, and 24 mo of age. Estimated marginal means (unadjusted means are presented in Tables 5 and 6), *P < 0.05, **P < 0.01 for impact on stunting of treatment group [FFR + CSB (*n* = 576); RFR + CSB (*n* = 575); NFR + CSB (*n* = 542); FFR + LNS (*n* = 550); FFR + MNP (*n* = 587)] compared with the control group (*n* = 574) (Table 4). CSB, corn-soy blend; FFR, full family ration; LNS, lipid-based nutrient supplement; MNP, micronutrient powder; NFR, no family ration; RFR, reduced family ration.

*PROCOMIDA* significantly reduced the prevalence of stunting at 24 mo by 11.1 percentage points (pp) in the FFR + CSB and 6.5 pp in the FFR + MNP group (**Table 4**, **Figure 3**). The positive program impacts were already apparent at 1 mo of age in these 2 groups (−5.1 pp and −3.8 pp for the FFR + CSB and FFR + MNP groups, respectively) and the size of the impact generally increased with age, more than doubling between 1 and 24 mo in the FFR + CSB group and increasing by ∼70% in the FFR + MNP group in the same period. The differences between these 2 groups and the control group were also statistically significant at 9 and 12 mo. At 1 mo there was also a significant program impact on stunting in the RFR + CSB group but this impact was not maintained past this age.

**TABLE 4:** Impact of *PROCOMIDA* treatment groups compared with control on growth of Guatemalan children^1^

	FFR + CSB	RFR + CSB	NFR + CSB	FFR + LNS	FFR + MNP
	(*n* = 576)	(*n* = 575)	(*n* = 542)	(*n* = 550)	(*n* = 587)
Stunted (length-for-age *z* score <−2)					
1 mo	−5.05*	−4.06*	−1.38	−1.39	−3.82*
4 mo	−2.03	−2.18	−0.63	−2.52	−3.21
6 mo	−4.36	−2.68	0.02	−1.28	−4.20
9 mo	−5.60*	0.87	1.95	−0.63	−5.83*
12 mo	−6.56*	0.83	−3.42	−2.28	−5.81*
18 mo	−6.15	1.69	−3.04	−3.74	−3.20
24 mo	−11.10**	−1.42	−4.25	−3.00	−6.54*
Length-for-age *z* score					
1 mo	0.13 ± 0.07*	0.08 ± 0.06	−0.03 ± 0.07	−0.01 ± 0.08	0.03 ± 0.06
4 mo	0.08 ± 0.07	0.02 ± 0.06	0.01 ± 0.07	−0.01 ± 0.07	0.06 ± 0.06
6 mo	0.05 ± 0.07	0.01 ± 0.07	−0.03 ± 0.07	−0.04 ± 0.07	0.05 ± 0.06
9 mo	0.03 ± 0.07	−0.06 ± 0.07	−0.05 ± 0.07	−0.07 ± 0.07	0.03 ± 0.07
12 mo	0.09 ± 0.07	−0.07 ± 0.08	−0.00 ± 0.08	−0.05 ± 0.07	0.06 ± 0.07
18 mo	0.11 ± 0.07	−0.05 ± 0.07	0.01 ± 0.08	−0.03 ± 0.07	0.04 ± 0.07
24 mo	0.19 ± 0.08*	−0.01 ± 0.08	0.06 ± 0.08	−0.01 ± 0.07	0.11 ± 0.07
Length-for-age difference					
1 mo	0.24 ± 0.14*	0.12 ± 0.13	−0.08 ± 0.15	−0.03 ± 0.16	0.05 ± 0.11
4 mo	0.17 ± 0.15	0.03 ± 0.14	0.01 ± 0.15	−0.02 ± 0.15	0.12 ± 0.13
6 mo	0.11 ± 0.16	0.00 ± 0.15	−0.07 ± 0.15	−0.09 ± 0.15	0.10 ± 0.14
9 mo	0.06 ± 0.17	−0.15 ± 0.16	−0.13 ± 0.18	−0.18 ± 0.16	0.06 ± 0.16
12 mo	0.23 ± 0.17	−0.16 ± 0.20	−0.01 ± 0.19	−0.14 ± 0.18	0.15 ± 0.18
18 mo	0.32 ± 0.21	−0.13 ± 0.21	0.03 ± 0.22	−0.08 ± 0.19	0.13 ± 0.19
24 mo	0.59 ± 0.24*	−0.02 ± 0.24	0.21 ± 0.26	−0.04 ± 0.22	0.38 ± 0.22*

^1^Values are coefficients ± SEMs or pp from linear mixed model, test of simple effects comparing each treatment group with control (*n* = 574), *P < 0.05, ** *P *< 0.01. CSB, corn-soy blend; FFR, full family ration; LNS, lipid-based nutrient supplement; MNP, micronutrient powder; NFR, no family ration; pp, percentage point; RFR, reduced family ration.

Consistent with the program impact on stunting at 24 mo, *PROCOMIDA* also significantly reduced LAD and increased LAZ in the FFR + CSB group (0.59 cm and 0.19 for LAD and LAZ, respectively) and reduced LAD in the FFR + MNP group (0.38 cm) compared with the control group (Table 4). Program impacts on LAD and LAZ were also significant at 1 mo in the FFR + CSB compared with the control group, but not in the FFR + MNP group. There were no significant effects on LAD or LAZ in the other treatment groups compared with control at any individual time point (Table 4).

The FFR conferred greater benefits for children's linear growth compared with the RFR or NFR. Specifically, the prevalence of stunting was 6.5, 7.4, and 9.7 pp higher at 9, 12, and 24 mo in the RFR + CSB group and 7.5 pp higher at 9 mo in the NFR + CSB group than in the FFR + CSB group. In addition, LAZ and LAD were significantly higher in the FFR + CSB group than in the RFR + CSB group at 12, 18, and 24 mo (**Table 5**).

**TABLE 5:** Differential impacts of providing the FFR compared with the RFR or NFR as the family ration size in the *PROCOMIDA* program on Guatemalan children's growth^1^

	FFR + CSB	RFR + CSB		NFR + CSB	
	(*n* = 576)	(*n* = 575)	Impact estimate (RFR vs. FFR)^2^	(*n* = 542)	Impact estimate (NFR vs. FFR)^2^
Stunted (length-for-age *z* score <−2)					
1 mo	14.58	14.98	1.00	17.25	3.67
4 mo	19.06	18.29	−0.15	19.56	1.40
6 mo	22.36	23.48	1.68	25.83	4.37
9 mo	28.77	34.67	6.47*	35.42	7.54*
12 mo	37.91	44.87	7.39*	40.48	3.14
18 mo	53.57	61.22	7.85	56.09	3.11
24 mo	50.00	59.16	9.68*	56.32	6.85
Length-for-age *z* score					
1 mo	−0.99 ± 1.00	−1.02 ± 1.00	−0.05 ± 0.07	−1.11 ± 1.06	−0.16 ± 0.08
4 mo	−1.14 ± 1.09	−1.18 ± 1.04	−0.06 ± 0.07	−1.18 ± 1.04	−0.07 ± 0.07
6 mo	−1.34 ± 1.01	−1.37 ± 1.00	−0.04 ± 0.07	−1.39 ± 0.99	−0.08 ± 0.07
9 mo	−1.58 ± 0.98	−1.65 ± 0.99	−0.09 ± 0.07	−1.63 ± 0.99	−0.08 ± 0.08
12 mo	−1.74 ± 0.96	−1.89 ± 0.97	−0.16 ± 0.08*	−1.82 ± 1.00	−0.09 ± 0.07
18 mo	−2.07 ± 0.91	−2.23 ± 0.92	−0.16 ± 0.07*	−2.16 ± 0.96	−0.10 ± 0.08
24 mo	−2.04 ± 0.89	−2.23 ± 0.93	−0.20 ± 0.08**	−2.16 ± 0.97	−0.13 ± 0.08
Length-for-age-difference					
1 mo	−1.94 ± 1.96	−1.99 ± 1.96	−0.12 ± 0.15	−2.18 ± 2.08	−0.33 ± 0.17
4 mo	−2.42 ± 2.31	−2.50 ± 2.19	−0.14 ± 0.15	−2.52 ± 2.20	−0.16 ± 0.16
6 mo	−2.95 ± 2.21	−2.99 ± 2.18	−0.10 ± 0.16	−3.06 ± 2.17	−0.18 ± 0.16
9 mo	−3.66 ± 2.25	−3.82 ± 2.26	−0.21 ± 0.16	−3.79 ± 2.30	−0.20 ± 0.18
12 mo	−4.30 ± 2.35	−4.65 ± 2.37	−0.39 ± 0.19*	−4.50 ± 2.47	−0.24 ± 0.18
18 mo	−5.79 ± 2.50	−6.22 ± 2.55	−0.45 ± 0.21*	−6.05 ± 2.69	−0.29 ± 0.22
24 mo	−6.49 ± 2.77	−7.05 ± 2.89	−0.61 ± 0.24**	−6.83 ± 3.03	−0.38 ± 0.26

^1^Values are percentages of means ± SDs unless otherwise indicated. CSB, corn-soy blend; FFR, full family ration; NFR, no family ration; RFR, reduced family ration.

^2^Values are coefficients ± SEMs from linear mixed model, test of simple effects comparing RFR + CSB and NFR + CSB with FFR + CSB. *P < 0.05, **P < 0.01.

We also found significantly different program impacts by type of micronutrient-fortified individual ration (provided along with the FFR). Both CSB and MNP were effective at reducing stunting and LAD and increasing LAZ. LNS, on the other hand, was not (Table 4). In addition, LNS was significantly less effective at improving child growth outcomes as compared with CSB or MNP, although the difference between LNS and MNP was limited to the difference in LAD at 24 mo (**Table 6**). Among children who received the FFR, the prevalence of stunting at 24 mo was 8.1 pp higher for those who received LNS compared with CSB as an individual ration; and LAD was higher and LAZ lower at 12, 18, and 24 mo. Program impacts on stunting, LAZ, and LAD were generally larger in the CSB group than in the MNP group, but the differences were not statistically significant for any indicator at any time point.

**TABLE 6:** Differential impacts of providing CSB, LNS, or MNP as the individual ration in the *PROCOMIDA* program on Guatemalan children's growth^1^

	FFR + CSB	FFR + LNS	Impact estimate	FFR + MNP	Impact estimate	Impact estimate
	(*n *= 576)	(*n* = 550)	(LNS vs. CSB)^2^	(*n* = 587)	(MNP vs. CSB)^2^	(MNP vs. LNS)^2^
Stunted (length-for-age *z* score <−2)						
1 mo	14.58	18.30	3.66	14.82	1.23	−2.42
4 mo	19.06	18.51	−0.49	16.75	−1.19	−0.69
6 mo	22.36	25.41	3.07	21.27	0.16	−2.92
9 mo	28.77	33.70	4.97	27.35	−0.23	−5.20
12 mo	37.91	42.47	4.29	37.84	0.75	−3.54
18 mo	53.57	56.44	2.42	56.05	2.95	0.53
24 mo	50.00	58.29	8.12*	53.92	4.56	−3.55
Length-for-age *z* score						
1 mo	−0.99 ± 1.00	−1.12 ± 1.07	−0.14 ± 0.08	−1.05 ± 1.02	−0.10 ± 0.07	0.04 ± 0.07
4 mo	−1.14 ± 1.09	−1.22 ± 1.01	−0.09 ± 0.07	−1.12 ± 1.03	−0.02 ± 0.07	0.07 ± 0.07
6 mo	−1.34 ± 1.01	−1.42 ± 0.98	−0.09 ± 0.07	−1.29 ± 0.96	0.00 ± 0.07	0.09 ± 0.06
9 mo	−1.58 ± 0.98	−1.67 ± 1.00	−0.10 ± 0.07	−1.52 ± 0.97	0.01 ± 0.07	0.10 ± 0.07
12 mo	−1.74 ± 0.96	−1.89 ± 0.98	−0.14 ± 0.07*	−1.74 ± 0.97	−0.03 ± 0.07	0.12 ± 0.07
18 mo	−2.07 ± 0.91	−2.21 ± 0.98	−0.13 ± 0.07*	−2.12 ± 0.95	−0.06 ± 0.07	0.07 ± 0.06
24 mo	−2.04 ± 0.89	−2.24 ± 0.96	−0.20 ± 0.07**	−2.10 ± 0.91	−0.08 ± 0.07	0.12 ± 0.06
Length-for-age-difference						
1 mo	−1.94 ± 1.96	−2.20 ± 2.11	−0.27 ± 0.17	−2.06 ± 2.01	−0.20 ± 0.13	0.08 ± 0.14
4 mo	−2.42 ± 2.31	−2.60 ± 2.14	−0.19 ± 0.16	−2.38 ± 2.20	−0.05 ± 0.15	0.14 ± 0.14
6 mo	−2.95 ± 2.21	−3.12 ± 2.15	−0.20 ± 0.16	−2.85 ± 2.12	−0.00 ± 0.15	0.19 ± 0.14
9 mo	−3.66 ± 2.25	−3.88 ± 2.33	−0.24 ± 0.17	−3.56 ± 2.26	−0.00 ± 0.17	0.24 ± 0.17
12 mo	−4.30 ± 2.35	−4.67 ± 2.40	−0.37 ± 0.17*	−4.32 ± 2.41	−0.08 ± 0.16	0.29 ± 0.17
18 mo	−5.79 ± 2.50	−6.20 ± 2.72	−0.39 ± 0.19*	−5.95 ± 2.64	−0.18 ± 0.19	0.21 ± 0.18
24 mo	−6.49 ± 2.77	−7.11 ± 3.02	−0.63 ± 0.22**	−6.65 ± 2.86	−0.21 ± 0.22	0.42 ± 0.19*

^1^Values are percentages of means ± SDs unless otherwise indicated. CSB, corn-soy blend; FFR, full family ration; LNS, lipid-based nutrient supplement; MNP, micronutrient powder.

^2^Values are coefficients ± SEMs from linear mixed model, test of simple effects comparing FFR + LNS and FFR + MNP with FFR + CSB, and FFR + LNS with FFR + MNP. *P < 0.05, **P < 0.01.

Results from the robustness check (linear mixed-effects model with growth variables treated as missing if the measurement was taken >1 mo after the age time point) were in line with the results from the main analytic sample. The biggest differences between the 2 sets of analyses were found in the NFR + CSB group compared with the FFR + CSB group. In the analysis where measurements taken outside of the 1-mo age window were set to missing, the size of the differences between the 2 groups in stunting and LAZ was larger and significantly different at most time points. This analysis confirms the impacts in the FFR + CSB and FFR + MNP groups compared with control and further illustrates the relative effectiveness of the FFR + CSB group compared with the NFR + CSB group (**Supplemental Tables 5–7**).

## Discussion

*PROCOMIDA* significantly improved children's linear growth and reduced stunting. Positive program impacts were evident at 1 mo of age and increased over time up to 24 mo of age among children in the groups that received the FFR with CSB or MNP. In the FFR + CSB and FFR + MNP groups the effect by 24 mo represented an 18.3% and 10.8% decrease in the prevalence of stunting, respectively. With ∼30 mo of program exposure for each child (6 mo of pregnancy and 24 mo postpartum), this translates to a yearly stunting reduction of 7.3% and 4.3% in these 2 arms, which is well above the World Health Assembly target of reducing stunting by ∼3.9%/y (30). In addition, the size of the effect at 24 mo in the FFR + CSB group (0.19 LAZ) was larger than the standardized mean differences in LAZ found in a recent systematic review and meta-analysis: for nutrition education programs in food-secure populations the authors found a mean effect of 0.11 LAZ; the mean effect of complementary feeding interventions with or without nutrition education in food-insecure populations was estimated to be 0.08 LAZ (7).

There was no significant program impact on children's growth in the groups that received NFR (and CSB as their individual ration), or the FFR with LNS. In addition, only the effect on stunting at 1 mo was statistically significant in the group that received the RFR. These results indicate that the provision of the FFR with either CSB or MNP as individual rations was effective at reducing stunting during the first 1000 d. The positive program impacts apparent at 1 mo suggest an important role for the PM2A program during pregnancy and early lactation. However, the increase in size of these positive program impacts at 24 mo indicates that the provision of the full PM2A package for the entire 1000-d period is essential for maintaining and maximizing program impacts on child linear growth.

This is the first study that demonstrates a significant impact of MNP on reducing stunting when given in the context of a FA-MCHN program that provided a monthly family food ration combined with health, nutrition, and hygiene BCC. Some of the studies that compared LNS and MNP showed impacts of LNS, but not of MNP, on linear growth (12, 31). However, a key limitation of these studies is that the MNP supplements used have not included some macrominerals known to be important for growth (e.g., calcium, magnesium, phosphorus, and potassium) whereas they are typically included in LNS (32). This has led researchers to conclude that MNPs were effective at reducing anemia as originally formulated, but not at improving linear growth (33). One of the potential reasons for not including macrominerals in previous MNP formulations (in addition to the fact that they were originally designed to address anemia and not growth) is the potential to affect taste and texture. In this study, we specifically designed the MNP and LNS to have the same micronutrient formulations so that the only differences between the supplements were the amounts of calories and lipids. To address the potential taste and texture issues with including the macrominerals in the MNP, we conducted acceptability trials with the supplements before the start of implementation and tested both a single dose and a split MNP dose. The split dose was rated higher than the single dose on perceived taste, smell, consistency, and appearance, and had similar acceptability ratings as the LNS (22). With these formulations, we found a positive impact on stunting apparent at 1 mo and a larger impact at 24 mo of age in the group that received MNP and no effect in the group that received LNS. A recent study from Bangladesh that provided MNP to full-term, low birth weight children 6–12 mo of age in conjunction with general nutrition, health, and hygiene education (but not family ration) also demonstrated a significant impact on reducing stunting (OR = 0.35 at 12 mo) (11). The MNP formulation for the Bangladesh study included 22 micronutrients, many with identical or nearly identical concentrations as in our study. The inclusion of these additional micronutrients may explain the differential findings of the Bangladesh study and our study compared with older studies that have found no effect of MNP on linear growth of young children.

We had expected at least similar linear growth effects in the groups that received either the MNP or LNS as the individual supplement, given that the micronutrient composition of the 2 supplements was identical. However, no effects on child growth were found in the LNS group. To date, impacts of LNSs on children's linear growth have been mixed. Our null results are in line with those from studies conducted in Malawi (13, 34) and Burkina Faso (35) which also did not find an effect of LNSs on linear growth outcomes, and in Ghana where the impact of LNSs on birth length was limited to primiparous women (36). Thus, we cannot exclude the possibility that the lack of impact could be due to limitations of the efficacy of LNSs in the Guatemalan context. It is also possible that the null results in the LNS group (and not in the MNP group) are related to differences in the utilization of the supplements. Based on maternal report, the percentage who consumed and the frequency of consumption were comparable in the MNP and LNS groups at 18 and 24 mo and were reportedly higher in the LNS group at 9 and 12 mo (Supplemental Table 4). However, these measures do not account for quantity of the supplements consumed. In our formative research, children who had LNS mixed with banana ate ∼72% of the mixture, whereas children that had the same amount of banana mixed with MNP ate ∼90% of the mixture (37). Thus, it is possible that more of the MNP compared with LNS was consumed by the targeted children. It is also possible that LNS supplements were more likely to be shared with other siblings than the MNPs, especially because LNSs can be consumed directly from the package, whereas MNPs need to be sprinkled on food.

Another potential explanation for the impact of FFR + MNP, and not FFR + LNS, on growth is group differences in program impacts on maternal IYCF practices, including early breastfeeding and complementary feeding practices. Mothers in the FFR + MNP group, compared with the control group, were significantly more likely to have initiated breastfeeding within the first hour of birth (5 pp) and to have exclusively breastfed their children at 1 mo (4 pp). These effects were not seen in the FFR + LNS group (unpublished data). In addition, children in the FFR + MNP compared with the control group were more likely to have received a minimally acceptable diet at 18 and 24 mo (a difference of 7 and 12 pp, respectively; *P* < 0.05); again, no impact of the program on these practices was found among the FFR + LNS group (unpublished data). It is possible that the provision of the MNP, which had to be stirred into complementary foods, may have increased overall dietary intake in addition to the additional micronutrients provided by the MNP. We also found positive program impacts on personal and home hygiene in the FFR + MNP group (and in the FFR + CSB group), but not in the FFR + LNS group, which may have contributed to the positive impacts on linear growth. However, given that all groups received the same BCC and that there were no group differences in participation between these 2 groups, the findings of a differential impact on hygiene practices between groups are difficult to interpret.

The impact pathways described above were similar in the group that received the FFR with CSB as the individual ration. In this group, we also found positive program impacts for child feeding practices (e.g., exclusive breastfeeding and minimum dietary diversity) as well as for hygiene outcomes. However, our study design does not allow for disentangling the relative contributions of the different program components and to determine the extent to which the family rations, the fortified individual rations, the BCC, or the health-strengthening interventions contributed to the improvements in linear growth. Reduced exposure to aflatoxin-contaminated maize (through substitution of locally procured maize with aflatoxin-free ration foods) could have contributed to the growth impact. Observational studies in West Africa have found an association between aflatoxin exposure and linear growth retardation in utero and in infants and young children (38–41). Inferring causality from these studies, however, is challenging because it is difficult to separate the effects of aflatoxin exposure from other determinants of stunted linear growth such as inadequate dietary intake and infections (42). Finally, another factor that could have contributed to differential impacts across the groups receiving different individual ration types is program participation, but we did not find meaningful differences in program participation across the groups that received the FFR with either CSB, LNS, or MNP.

The size of the family ration, however, played an essential role in program participation, despite the limited problems of severe food insecurity in the region. Participation in the group that did not receive a family ration was lowest throughout the study. Lower participation meant a smaller proportion of eligible beneficiaries in this group having access to the individual food rations and attending the monthly BCC sessions which likely limited the potential effectiveness of the interventions provided by the program. In fact, the only significant program impact found in that group on IYCF or care practices was an increase in the proportion of children who were exclusively breastfed at 4 and 6 mo. The lack of impact on other IYCF and care indicators in the NFR + CSB group may have been due to lack of exposure to the messages through the BCC sessions. By contrast, in the FFR + CSB group, where program participation (and hence BCC participation) was high throughout the program period, positive impacts were found for exclusive breastfeeding, early initiation of breastfeeding, child dietary diversity, and hygiene practices (unpublished data). This suggests that with greater attendance at BCC sessions, women were more likely to adopt optimal IYCF and hygiene practices, which in turn may have contributed to the positive impacts on growth in this group. Positive impacts on IYCF and care practices were also found in the RFR + CSB group but were limited to improvements in early initiation of breastfeeding, exclusive breastfeeding at 4 and 6 mo, and minimum meal frequency.

This study used a rigorous cluster-randomized controlled study design and collected data along the hypothesized impact pathways to assess the plausibility of these pathways. However, a few limitations should be noted. First, we did not have a survey in late pregnancy. An assessment in late pregnancy would have given us better information on program participation and use of the individual rations during pregnancy. We were limited to how many survey rounds we could conduct and prioritized postbirth time points. Second, the study was designed as an intent-to-treat effectiveness study, and did not closely monitor supplement use or collect detailed diet or illness information which could have helped to better elucidate the program impact pathways. The study was also not designed to allow for an analysis of the contribution of the different program components (food, BCC, use of health services) to the impacts found. Doing so would have required many more comparison groups, which was not possible for financial and logistic reasons.

This study showed that it was possible to significantly improve linear growth and reduce stunting in the first 1000 d through a well-designed FA-MCHN program that targeted mothers during pregnancy and the child up to 24 mo of age. Unlike similar programs that target pregnant women or children <24 mo of age, the *PROCOMIDA* program specifically targeted and enrolled women during pregnancy and provided the package of interventions to the same mothers and families for the whole first 1000-d period. With this intensive and prolonged approach, the program achieved its main objective of reducing stunting. The fact that an impact on stunting could be detected as early as 1 mo of age highlights the importance of intervening during pregnancy (even in energy/food-secure populations) to achieve longer-term impacts on child stunting. The study also shows that the provision of a relatively large family ration (contributing 270 kcal/d per capita) in this type of program increased participation and effectiveness of the program. The evaluation also showed that in the context of this large FA-MCHN program, MNP and CSB, but not LNS, were effective in reducing stunting. Although the program was successful, the prevalence of stunting was still nearly 50% in the most effective treatment group at 24 mo. This indicates the need for additional complementary strategies to effectively address the multiple causes of stunting in this population to build on the program's successes.

## Supplementary Material

Supplemental Tables

## Notes

The study was made possible by the generous support of the American people through the support of the Office of Food for Peace, Bureau for Democracy, Conflict, and Humanitarian Assistance, and the Office of Health, Infectious Diseases, and Nutrition, Bureau for Global Health, US Agency for International Development (USAID), under terms of Cooperative Agreement No. AID-OAA-A-12-00005, through the Food and Nutrition Technical Assistance III Project, managed by FHI 360. Funding was also received from the CGIAR Research Program on Agriculture for Nutrition and Health led by the International Food Policy Research Institute.

Supplemental Tables 1–7 are available from the “Supplementary data” link in the online posting of the article and from the same link in the online table of contents at https://academic.oup.com/jn/.

Author disclosures: DKO, JL, LB, and MTR, no conflicts of interest.

Abbreviations used: BCC, behavior change communication; CSB, corn-soy blend; FA-MCHN, food-assisted maternal and child health and nutrition; FFP, Food for Peace; FFR, full family ration; IYCF, infant and young child feeding; LAD, length-for-age difference; LAZ, length-for-age Z score; LNS, lipid-based nutrient supplement; MNP, micronutrient powder; NFR, no family ration; PM2A, Preventing Malnutrition in children under 2 years of age Approach; pp, percentage point; RFR, reduced family ration; USAID, United States Agency for International Development.

## References

[1] Black RE, Victora CG, Walker SP, Bhutta ZA, Christian P, de Onis M, Ezzati M, Grantham-McGregor S, Katz J, Martorell R Maternal and child undernutrition and overweight in low-income and middle-income countries. Lancet 2013;382:427–51. 23746772 10.1016/S0140-6736(13)60937-X

[2] International Food Policy Research Institute (IFPRI) Global nutrition report 2016: from promise to impact: ending malnutrition by 2030. Washington (DC): International Food Policy Research Institute; 2016.

[3] Delgado HL Technical report. Status and trends in chronic malnutrition in Guatemala. Chevy Chase, MD: University Research Co., LLC; 2010.

[4] Schroeder DG, Martorell R, Rivera JA, Ruel MT, Habicht JP Age differences in the impact of nutritional supplementation on growth. J Nutr 1995;125:1051S–9S. 7722707 10.1093/jn/125.suppl_4.1051S

[5] Martorell R, Schroeder DG, Rivera JA, Kaplowitz HJ Patterns of linear growth in rural Guatemalan adolescents and children. J Nutr 1995;125:1060S–7S. 7722708 10.1093/jn/125.suppl_4.1060S

[6] Martorell R, Melgar P, Maluccio JA, Stein AD, Rivera JA The nutrition intervention improved adult human capital and economic productivity. J Nutr 2010;140:411–14. 20032473 10.3945/jn.109.114504 PMC6497421

[7] Panjwani A, Heidkamp R Complementary feeding interventions have a small but significant impact on linear and ponderal growth of children in low- and middle-income countries: a systematic review and meta-analysis. J Nutr 2017;147:2169S–78S. 28904113 10.3945/jn.116.243857

[8] Bhandari N, Bahl R, Taneja S Effect of micronutrient supplementation on linear growth of children. Br J Nutr 2001;85(Suppl 2):S131–7. 11509101

[9] Christian P, Shaikh S, Shamim AA, Mehra S, Wu L, Mitra M, Ali H, Merrill RD, Choudhury N, Parveen M Effect of fortified complementary food supplementation on child growth in rural Bangladesh: a cluster-randomized trial. Int J Epidemiol 2015;44:1862–76. 26275453 10.1093/ije/dyv155 PMC4689999

[10] Allen LH, Peerson JM, Olney DK Provision of multiple rather than two or fewer micronutrients more effectively improves growth and other outcomes in micronutrient-deficient children and adults. J Nutr 2009;139:1022–30. 19321586 10.3945/jn.107.086199

[11] Shafique S, Sellen DW, Lou W, Jalal CS, Jolly SP, Zlotkin SH Mineral- and vitamin-enhanced micronutrient powder reduces stunting in full-term low-birth-weight infants receiving nutrition, health, and hygiene education: a 2 × 2 factorial, cluster-randomized trial in Bangladesh. Am J Clin Nutr 2016;103:1357–69. 27053383 10.3945/ajcn.115.117770

[12] Adu-Afarwuah S, Lartey A, Okronipa H, Ashorn P, Peerson JM, Arimond M, Ashorn U, Zeilani M, Vosti S, Dewey KG Small-quantity, lipid-based nutrient supplements provided to women during pregnancy and 6 mo postpartum and to their infants from 6 mo of age increase the mean attained length of 18-mo-old children in semi-urban Ghana: a randomized controlled trial. Am J Clin Nutr 2016;104:797–808. 27534634 10.3945/ajcn.116.134692 PMC4997301

[13] Ashorn P, Alho L, Ashorn U, Cheung YB, Dewey KG, Harjunmaa U, Lartey A, Nkhoma M, Phiri N, Phuka J The impact of lipid-based nutrient supplement provision to pregnant women on newborn size in rural Malawi: a randomized controlled trial. Am J Clin Nutr 2015;101:387–97. 25646337 10.3945/ajcn.114.088617

[14] Imdad A, Bhutta ZA Maternal nutrition and birth outcomes: effect of balanced protein-energy supplementation. Paediatr Perinat Epidemiol 2012;26:178–90. 22742610 10.1111/j.1365-3016.2012.01308.x

[15] Haider BA, Bhutta ZA Multiple-micronutrient supplementation for women during pregnancy. In: Bhutta ZA, editor Cochrane database of systematic reviews. Chichester, UK: John Wiley & Sons, Ltd; 2015. 10.1002/14651858.CD004905.pub4 PMC6464025 26522344

[16] Lu W-P, Lu M-S, Li Z-H, Zhang C-X, Lind T Effects of multimicronutrient supplementation during pregnancy on postnatal growth of children under 5 years of age: a meta-analysis of randomized controlled trials. PLoS One 2014;9:e88496. 24586335 10.1371/journal.pone.0088496 PMC3930526

[17] Christian P, Kim J, Mehra S, Shaikh S, Ali H, Shamim AA, Wu L, Klemm R, Labrique AB, West KP Effects of prenatal multiple micronutrient supplementation on growth and cognition through 2 y of age in rural Bangladesh: the JiVitA-3 trial. Am J Clin Nutr 2016;104:1175–82. 27604769 10.3945/ajcn.116.135178

[18] Ruel MT, Menon P, Habicht J-P, Loechl C, Bergeron G, Pelto G, Arimond M, Maluccio J, Michaud L, Hankebo B Age-based preventive targeting of food assistance and behaviour change and communication for reduction of childhood undernutrition in Haiti: a cluster randomised trial. Lancet 2008;371:588–95. 18280329 10.1016/S0140-6736(08)60271-8

[19] Leroy JL, Olney D, Ruel M Tubaramure, a food-assisted integrated health and nutrition program, reduces child stunting in Burundi: a cluster-randomized controlled intervention trial. J Nutr 2018;148:445–52. 29546306 10.1093/jn/nxx063

[20] MSPAS; INE; ICF Encuesta Nacional de Salud Materno Infantil 2014-2015. Informe final. Guatemala City: MSPAS; INE; ICF; 2017.

[21] Richter S, Harris J, Leroy J, Olney D, Ruel M Strengthening and evaluating the “Preventing Malnutrition in children under two years of age Approach” (PM2A) in Guatemala. Washington (DC): Poverty, Health, and Nutrition Division, FHI 360/FANTA; 2011.

[22] Olney D, Arriola M, Carranza R, Leroy J, Richter S, Harris J, Ruel M, Becker E Report of formative research conducted in Alta Verapaz, Guatemala, to help inform the health-strengthening activities and the social and behavior change communication strategy that will be implemented through the Mercy Corps PM2A program – PROCOMIDA. Washington (DC): FHI 360/FANTA; 2012.

[23] Donegan S, Maluccio JA, Myers CK, Menon P, Ruel MT, Habicht J-P Two food-assisted maternal and child health nutrition programs helped mitigate the impact of economic hardship on child stunting in Haiti. J Nutr 2010;140:1139–45. 20392883 10.3945/jn.109.114272

[24] Cogill B Anthropometric indicators measurement guide. Washington (DC): Food and Nutrition Technical Assistance Project, Academy for Educational Development; 2003.

[25] Ballard T, Coates J, Swindale A, Deitchler M Household Hunger Scale (HHS): indicator definition and measurement guide FHI 360; 2011.

[26] World Health Organization Indicators for assessing infant and young child feeding practices: part II measurement. Geneva: World Health Organization; 2010.

[27] WHO Multicentre Growth Reference Study Group WHO child growth standards: length/height-for-age, weight-for-age, weight-for-length, weight-for-height and body mass index-for-age: methods and development. Geneva: World Health Organization; 2006.

[28] Leroy JL, Ruel M, Habicht J-P, Frongillo EA Linear growth deficit continues to accumulate beyond the first 1000 days in low- and middle-income countries: global evidence from 51 national surveys. J Nutr 2014;144:1460–6. 24944283 10.3945/jn.114.191981

[29] Filmer D, Pritchett LH Estimating wealth effects without expenditure data—or tears: an application to educational enrollments in states of India. Demography 2001;38:115–32. 11227840 10.1353/dem.2001.0003

[30] de Onis M, Dewey KG, Borghi E, Onyango AW, Blössner M, Daelmans B, Piwoz E, Branca F The World Health Organization's global target for reducing childhood stunting by 2025: rationale and proposed actions. Matern Child Nutr 2013;9:6–26. 10.1111/mcn.12075 PMC6860845 24074315

[31] Dewey KG, Mridha MK, Matias SL, Arnold CD, Cummins JR, Khan MSA, Maalouf-Manasseh Z, Siddiqui Z, Ullah MB, Vosti SA Lipid-based nutrient supplementation in the first 1000 d improves child growth in Bangladesh: a cluster-randomized effectiveness trial. Am J Clin Nutr 2017;105:944–57. 28275125 10.3945/ajcn.116.147942

[32] Arimond M, Zeilani M, Jungjohann S, Brown KH, Ashorn P, Allen LH, Dewey KG Considerations in developing lipid-based nutrient supplements for prevention of undernutrition: experience from the International Lipid-Based Nutrient Supplements (iLiNS) project. Matern Child Nutr 2015;11:31–61. 10.1111/mcn.12049 PMC6860325 23647784

[33] De-Regil LM, Suchdev PS, Vist GE, Walleser S, Peña-Rosas JP Home fortification of foods with multiple micronutrient powders for health and nutrition in children under two years of age (review). Evid Based Child Health 2013;8:112–201. 23878126 10.1002/ebch.1895

[34] Thakwalakwa CM, Ashorn P, Jawati M, Phuka JC, Cheung YB, Maleta KM An effectiveness trial showed lipid-based nutrient supplementation but not corn–soya blend offered a modest benefit in weight gain among 6- to 18-month-old underweight children in rural Malawi. Public Health Nutr 2012;15:1755–62. 22691922 10.1017/S1368980012003023

[35] Lanou H, Huybregts L, Roberfroid D, Nikièma L, Kouanda S, Van Camp J, Kolsteren P Prenatal nutrient supplementation and postnatal growth in a developing nation: an RCT. Pediatrics 2014;133:e1001–8. 24590752 10.1542/peds.2013-2850

[36] Adu-Afarwuah S, Lartey A, Okronipa H, Ashorn P, Zeilani M, Peerson JM, Arimond M, Vosti S, Dewey KG Lipid-based nutrient supplement increases the birth size of infants of primiparous women in Ghana. Am J Clin Nutr 2015;101:835–46. 25833980 10.3945/ajcn.114.091546

[37] Olney DK, Richter S, Becker E, Roopnaraine T, Margolies A, Kennedy A, Leroy JL, Ruel MT A process evaluation of the PROCOMIDA “Preventing Malnutrition in children under 2 Approach” in Guatemala. Washington (DC): FHI 360; 2013.

[38] Shuaib FMB, Jolly PE, Ehiri JE, Yatich N, Jiang Y, Funkhouser E, Person SD, Wilson C, Ellis WO, Wang J-S Association between birth outcomes and aflatoxin B_1_ biomarker blood levels in pregnant women in Kumasi, Ghana. Trop Med Int Heal 2010;15:160–7. 10.1111/j.1365-3156.2009.02435.x 20003033

[39] Turner PC, Collinson AC, Cheung YB, Gong Y, Hall AJ, Prentice AM, Wild CP Aflatoxin exposure in utero causes growth faltering in Gambian infants. Int J Epidemiol 2007;36:1119–25. 17576701 10.1093/ije/dym122

[40] Gong YY, Cardwell K, Hounsa A, Egal S, Turner PC, Hall AJ, Wild CP Dietary aflatoxin exposure and impaired growth in young children from Benin and Togo: cross sectional study. BMJ 2002;325:20–1. 12098724 10.1136/bmj.325.7354.20 PMC116667

[41] Gong Y, Hounsa A, Egal S, Turner PC, Sutcliffe AE, Hall AJ, Cardwell K, Wild CP Postweaning exposure to aflatoxin results in impaired child growth: a longitudinal study in Benin, West Africa. Environ Health Perspect 2004;112:1334–8. 15345349 10.1289/ehp.6954 PMC1247526

[42] Wild CP, Miller JD, Groopman JD Mycotoxin control in low- and middle-income countries. Geneva: World Health Organization; 2016. 27030861

