# Supplementary material for: PROCOMIDA, a Food-Assisted Maternal and Child Health and Nutrition Program, Reduces Child Stunting in Guatemala: A Cluster-Randomized Controlled Intervention Trial
Source: J Nutr. 2018 Aug 31;148(9):1493–505. doi: 10.1093/jn/nxy138 (PMC6118165; doi:10.1093/jn/nxy138)
Supplement: Supplemental Tables [file nxy138_supplemental_tables.docx]

**Supplementary data**

**Supplemental Table 1** Composition of family food rations

|  | Full family food ration (FFR) | |  | Reduced family food ration (RFR) | |
| --- | --- | --- | --- | --- | --- |
|  | Weight (kg) | Energy (kcal) |  | Weight (kg) | Energy (kcal) |
| Rice | 6.00 | 21,600 |  | 3.000 | 10,800 |
| Beans | 4.00 | 13,600 |  | 3.000 | 10,200 |
| Vegetable oil | 1.85 | 16,354 |  | 0.925 | 8,177 |
| Total | 11.85 | 51,554 |  | 6.925 | 29,177 |
| Total kcal/d per capita^1^ |  | 269^2^ |  |  | 152^2^ |

^1^ Total kcal/d per capita was calculated using an average household size of 6.3 members (the average household size in the enrollment survey) and 30.42 days/month. ^2^ The individual ration was not meant to be shared, so it is not included in the computation of the total kcal/d per capita. If the individual CSB ration was shared, it would provide an additional 78 kcal/d per capita.

**Supplementary data**

**Supplemental Table 2** Enrollment characteristics of Guatemalan households and mothers included in the study sample and those that attrited ^1^

|  | Ever attrited | Included in the study sample |
| --- | --- | --- |
|  | (*n* = 1141) | (*n* = 3404) |
| *Household and housing characteristics* |  |  |
| Size | 6.4±3.0* | 6.2±3.0 |
| Owns home | 96.6 | 97.7 |
| Has dirt floor | 83.4 | 82.2 |
| Has wood walls | 68.7 | 82.2 |
| Moderate/severe hunger | 7.5 | 8.0 |
| *Household heads' characteristics* |  |  |
| Age (y) | 40.4±14.0* | 39.4±13.8 |
| Male (%) | 93.7 | 94.0 |
| Is indigenous (%) | 99.3 | 99.7 |
| Speaks Spanish (%) | 46.7 | 44.4 |
| Has not had any education or only preschool (%) | 49.4* | 45.3 |
| Occupation |  |  |
| Agriculture on own or family land (%) | 55.8 | 57.3 |
| Agriculture laborer (%) | 21.4 | 23.1 |
| *Mothers' characteristics* |  |  |
| Age (y) | 24.8±7.0 | 24.8±6.4 |
| Married/relationship & living with husband/partner (%) | 95.7 | 96.1 |
| Is indigenous (%) | 99.0 | 99.4 |
| Speaks Spanish (%) | 31.5 | 32.0 |
| Has not had any education or only preschool (%) | 37.1* | 33.3 |
| Height (cm) | 146.6±4.7 | 146.8±4.6 |

^1^ All values are mean ± SD or percentages unless otherwise indicated. Different from the households and mothers included in the study sample, * *P* < 0.05.

**Supplementary data**

**Supplemental Table 3** Use of corn soy blend (CSB) by mothers in the groups that received CSB at 4 and 6 mo postpartum and among children 6, 9, 12, 18 and 24 mo of age

|  | FFR+CSB^1^ | RFR+CSB^1^ | Difference^2^ | NFR+CSB^1^ | Difference^2^ |
| --- | --- | --- | --- | --- | --- |
|  | (*n* = 576) | (*n* = 575) | (FFR vs. RFR) | (*n* = 541) | (FFR vs. NFR) |
| CSB |  |  |  |  |  |
| Mother (past 24 hours) |  |  |  |  |  |
| 4 mo | 44.21 | 37.02 | -7.26 | 23.22 | 21.04** |
| 6 mo | 41.78 | 34.27 | -7.49 | 20.07 | 21.70** |
| Mother (number of days in last week) |  |  |  |  |  |
| 4 mo | 2.16±2.47 | 1.81±2.33 | -0.35±0.31 | 1.06±1.93 | -1.09±0.27** |
| 6 mo | 1.99±2.25 | 1.67±2.13 | -0.32±0.22 | 1.01±1.86 | -0.98±0.22** |
| Child (past 24 hours) |  |  |  |  |  |
| 6 mo | 20.97 | 16.51 | -4.52 | 14.63 | -6.73** |
| 9 mo | 40.28 | 36.24 | -4.02 | 23.25 | -17.03** |
| 12 mo | 38.78 | 36.70 | -2.06 | 22.51 | -16.25** |
| 18 mo | 43.75 | 40.17 | -3.58 | 26.38 | -17.37** |
| 24 mo | 38.37 | 34.43 | -3.93 | 17.53 | -20.84** |
| Child (number of days in last week) |  |  |  |  |  |
| 6 mo | 0.96±1.96 | 0.73±1.72 | -0.21±0.21 | 0.54±1.46 | -0.44±0.17* |
| 9 mo | 2.03±2.52 | 1.73±2.27 | -0.30±0.24 | 1.09±1.99 | -0.94±0.22** |
| 12 mo | 1.99±2.41 | 1.79±2.37 | -0.19±0.19 | 1.11±2.01 | -0.88±0.19** |
| 18 mo | 2.08±2.42 | 1.96±2.35 | -0.12±0.20 | 1.17±2.10 | -0.91±0.23** |
| 24 mo | 1.71±2.21 | 1.43±2.03 | -0.28±0.18 | 0.88±1.83 | -0.83±0.23** |

^1^ Values are % or mean ± SD. ^2^ Values are coefficient ± SEM from linear mixed model, test of simple effects comparing CSB to LNS use; CSB to MNP use, and; LNS to MNP use. **P* < 0.05 or ** *P* < 0.01. CSB, corn-soy blend; FFR, full family ration; LNS, lipid-based nutrient supplement; MNP, micronutrient powder.

**Supplementary data**

**Supplemental Table 4** Use of corn soy blend (CSB), lipid-based nutrient supplement (LNS) and micronutrient powder (MNP) on the groups that received the full family ration by pregnant and lactating women during pregnancy and up to 6 mo postpartum and among children 6, 9, 12, 18 and 24 mo

|  | FFR+CSB^1^ | FFR+LNS^1^ | Difference^2^ | FFR+MNP^1^ | Difference^2^ | Difference^2^ |
| --- | --- | --- | --- | --- | --- | --- |
|  | (CSB use) | (LNS use) | (CSB vs. LNS) | (MNP use) | (CSB vs. MNP) | (LNS vs. MNP) |
|  | (*n* = 576) | (*n* = 549) |  | (*n* = 587) |  |  |
| Mother |  |  |  |  |  |  |
| Enrollment (ever during pregnancy) | - | 18.76 | - | 10.73 | - |  |
| 1 mo (during pregnancy, recall at 1 mo) | - | 41.89 | - | 43.00 | - |  |
| 1 mo (past 24 hours) | - | 38.62 |  | 39.25 |  | 0.75 |
| 4 mo (past 24 hours) | 44.21 | 58.47 | 14.26* | 63.48 | 19.50** | 5.24 |
| 6 mo (past 24 hours) | 41.78 | 64.30 | 22.51** | 66.95 | 25.36** | 2.86 |
| Child (in the past 24 hours) |  |  |  |  |  |  |
| 6 mo | 20.97 | 2.60 | -19.70** | 0.93 | -21.32** | -1.62 |
| 9 mo | 40.28 | 65.82 | 25.68** | 51.11 | 10.87* | -14.81** |
| 12 mo | 38.78 | 64.36 | 25.76** | 57.75 | 19.04** | -6.71 |
| 18 mo | 43.75 | 65.64 | 22.02** | 63.03 | 19.32** | -2.70 |
| 24 mo | 38.37 | 60.36 | 22.13** | 56.22 | 17.89** | -4.24 |
| Child (number of days in the past week) |  |  |  |  |  |  |
| 6 mo | 0.96±1.96 | 0.08±0.70 | -0.99±0.16** | 0.0±0.67 | -0.94±0.16** | 0.05±0.09 |
| 9 mo | 2.03±2.52 | 4.22±3.14 | 2.19±0.27** | 3.13±3.08 | 1.10±0.29** | -1.08±0.30** |
| 12 mo | 1.99±2.41 | 4.15±3.16 | 2.16±0.27** | 3.50±3.07 | 1.52±0.25** | -0.64±0.31* |
| 18 mo | 2.08±2.42 | 4.35±3.11 | 2.27±0.27** | 3.87±3.01 | 1.79±0.25** | -0.47±0.30 |
| 24 mo | 1.71±2.21 | 3.90±3.19 | 2.19±0.27** | 3.54±3.04 | 1.83±0.24** | -0.36±0.28 |

^1^ Values are % or mean ± SD. ^2^ Values are coefficient ± SEM from linear mixed model, test of simple effects comparing CSB to LNS use; CSB to MNP use, and; LNS to MNP use. **P* < 0.05 or ** *P* < 0.01. CSB, corn-soy blend; FFR, full family ration; LNS, lipid-based nutrient supplement; MNP, micronutrient powder.

**Supplementary data**

**Supplemental Table 5** Robustness check for impact of *PROCOMIDA* treatment groups compared to control on growth of Guatemalan children^1^

|  | FFR+CSB | RFR+CSB | NFR+CSB | FFR+LNS | FFR+MNP |
| --- | --- | --- | --- | --- | --- |
| Stunted (length-for-age Z-score < -2) |  |  |  |  |  |
| 1 mo | -5.80* | -4.43* | -1.57 | -2.23 | -3.21 |
| 4 mo | -3.46 | -3.30 | -0.10 | -2.80 | -3.60 |
| 6 mo | -4.97* | -4.56 | 0.54 | -1.33 | -4.77* |
| 9 mo | -5.92* | -0.54 | 2.72 | -1.02 | -5.30* |
| 12 mo | -6.83* | -1.44 | -1.70 | -1.29 | -5.88* |
| 18 mo | -6.10* | 1.71 | -1.19 | -2.10 | -2.37 |
| 24 mo | -10.65** | -1.80 | -2.22 | -1.26 | -5.86* |
| Length-for-age Z-score |  |  |  |  |  |
| 1 mo | 0.18±0.07** | 0.12±0.06* | 0.02±0.07 | 0.01±0.08 | 0.05±0.06 |
| 4 mo | 0.12±0.07* | 0.07±0.06 | 0.02±0.06 | -0.00±0.06 | 0.07±0.06 |
| 6 mo | 0.09±0.07 | 0.07±0.06 | -0.02±0.06 | -0.03±0.06 | 0.06±0.06 |
| 9 mo | 0.06±0.07 | -0.01±0.06 | -0.06±0.07 | -0.08±0.06 | 0.02±0.07 |
| 12 mo | 0.12±0.06* | -0.01±0.07 | -0.01±0.07 | -0.07±0.07 | 0.06±0.07 |
| 18 mo | 0.11±0.07 | -0.03±0.07 | -0.02±0.08 | -0.07±0.07 | 0.03±0.07 |
| 24 mo | 0.19±0.07** | -0.01±0.07 | 0.03±0.08 | -0.04±0.07 | 0.09±0.07 |
| Length-for-age difference |  |  |  |  |  |
| 1mo | 0.34±0.14** | 0.21±0.14 | -0.00±0.15 | 0.01±0.15 | 0.09±0.12 |
| 4 mo | 0.24±0.14* | 0.13±0.12 | 0.01±0.14 | -0.02±0.13 | 0.13±0.12 |
| 6 mo | 0.20±0.15 | 0.14±0.14 | -0.07±0.14 | -0.08±0.14 | 0.11±0.13 |
| 9 mo | 0.13±0.15 | -0.02±0.14 | -0.16±0.17 | -0.19±0.14 | 0.04±0.16 |
| 12 mo | 0.30±0.16* | -0.02±0.19 | -0.03±0.19 | -0.17±0.16 | 0.14±0.17 |
| 18 mo | 0.32±0.19* | -0.06±0.19 | -0.04±0.22 | -0.18±0.18 | 0.09±0.19 |
| 24 mo | 0.57±0.23** | 0.04±0.22 | 0.11±0.26 | -0.13±0.21 | 0.29±0.21 |

^1^ Values are coefficient ± SEM from linear mixed model, test of simple effects comparing each treatment group to control. Models used imputed values for growth measurements taken more than one month after an individual age time point. **P* < 0.05, ** *P* < 0.01. CSB, corn-soy blend; FFR, full family ration; LNS, lipid-based nutrient supplement; MNP, micronutrient powder; NFR, no family ration; RFR, reduced family ration.

**Supplementary data**

**Supplemental Table 6** Robustness check for differential impact estimates of reduced or no family ration compared to the full family ration provided in the *PROCOMIDA* program on growth of Guatemalan children^1^

|  | FFR+CSB^1^ | RFR+CSB^1^ | Impact estimate^2^ | NFR+CSB^1^ | Impact estimate^2^ |
| --- | --- | --- | --- | --- | --- |
|  |  |  | (RFR vs. FFR) |  | (NFR vs. FFR) |
| Stunted (length-for-age Z-score < -2) |  |  |  |  |  |
| 1 mo | 14.58 | 14.98 | 1.37 | 17.25 | 4.23* |
| 4 mo | 19.06 | 18.29 | 0.16 | 19.56 | 3.36 |
| 6 mo | 22.36 | 23.48 | 0.41 | 25.83 | 5.51* |
| 9 mo | 28.77 | 34.67 | 5.38* | 35.42 | 8.64* |
| 12 mo | 37.91 | 44.87 | 5.39* | 40.48 | 5.13 |
| 18 mo | 53.57 | 61.22 | 7.81* | 56.09 | 4.91 |
| 24 mo | 50.00 | 59.16 | 8.84* | 56.32 | 8.43* |
| Length-for-age Z-score |  |  |  |  |  |
| 1 mo | -0.99±1.00 | -1.02±1.00 | -0.06±0.07 | -1.11±1.06 | -0.17±0.08* |
| 4 mo | -1.14±1.09 | -1.18±1.04 | -0.04±0.07 | -1.18±1.04 | -0.10±0.07 |
| 6 mo | -1.34±1.01 | -1.37±1.00 | -0.02±0.07 | -1.39±0.99 | -0.12±0.07* |
| 9 mo | -1.58±0.98 | -1.65±0.99 | -0.06±0.06 | -1.63±0.99 | -0.12±0.08 |
| 12 mo | -1.74±0.96 | -1.89±0.97 | -0.13±0.07* | -1.82±1.00 | -0.13±0.07* |
| 18 mo | -2.07±0.91 | -2.23±0.92 | -0.14±0.07* | -2.16±0.96 | -0.13±0.08* |
| 24 mo | -2.04±0.89 | -2.23±0.93 | -0.17±0.08** | -2.16±0.97 | -0.16±0.08* |
| Length-for-age difference |  |  |  |  |  |
| 1 mo | -1.94±1.96 | -1.99±1.96 | -0.13±0.14 | -2.18±2.08 | -0.34±0.16* |
| 4 mo | -2.42±2.31 | -2.50±2.19 | -0.11±0.15 | -2.52±2.20 | -0.23±0.16 |
| 6 mo | -2.95±2.21 | -2.99±2.18 | -0.05±0.14 | -3.06±2.17 | -0.27±0.15 |
| 9 mo | -3.66±2.25 | -3.82±2.26 | -0.15±0.15 | -3.79±2.30 | -0.29±0.18 |
| 12 mo | -4.30±2.35 | -4.65±2.37 | -0.31±0.17 | -4.50±2.47 | -0.33±0.17 |
| 18 mo | -5.79±2.50 | -6.22±2.55 | -0.38±0.18* | -6.05±2.69 | -0.36±0.21 |
| 24 mo | -6.49±2.77 | -7.05±2.89 | -0.53±0.21* | -6.83±3.03 | -0.46±0.25 |

^1^ Values are % or mean ± SD. ^2^ Values are coefficient ± SEM from linear mixed model, test of simple effects comparing RFR+CSB and NFR+CSB to FFR+CSB. Models used imputed values for growth measurements taken more than one month after an individual age time point. **P* < 0.05, ** *P* < 0.01. CSB, corn-soy blend; FFR, full family ration; LNS, lipid-based nutrient supplement; MNP, micronutrient powder; NFR, no family ration; RFR, reduced family ration.

**Supplementary data**

**Supplemental Table 7** Robustness check for providing corn-soy blend (CSB), lipid-based nutrient supplement (LNS) or micronutrient powder (MNP) as the individual ration in the *PROCOMIDA* program on growth of Guatemalan children^1^

|  | FFR+CSB^1^ | FFR+LNS^1^ | Impact estimate | FFR+MNP^1^ | Impact estimate | Impact estimate |
| --- | --- | --- | --- | --- | --- | --- |
|  |  |  | (LNS vs. CSB)^2^ |  | (MNP vs. CSB)^2^ | (MNP vs. LNS)^2^ |
| Stunted (length-for-age Z-score < -2) |  |  |  |  |  |  |
| 1 mo | 14.58 | 18.30 | 3.57* | 14.82 | 2.59 | -0.98 |
| 4 mo | 19.06 | 18.51 | 0.66 | 16.75 | -0.14 | -0.80 |
| 6 mo | 22.36 | 25.41 | 3.64 | 21.27 | 0.20 | -3.43 |
| 9 mo | 28.77 | 33.70 | 4.90 | 27.35 | 0.62 | -4.23 |
| 12 mo | 37.91 | 42.47 | 5.54* | 37.84 | 0.95 | -4.59 |
| 18 mo | 53.57 | 56.44 | 4.01 | 56.05 | 3.73 | -0.28 |
| 24 mo | 50.00 | 58.29 | 9.39** | 53.92 | 4.78 | -4.60 |
| Length-for-age Z-score |  |  |  |  |  |  |
| 1 mo | -0.99±1.00 | -1.12±1.07 | -0.17±0.08* | -1.05±1.02 | -0.13±0.07 | 0.05±0.07 |
| 4 mo | -1.14±1.09 | -1.22±1.01 | -0.12±0.07 | -1.12±1.03 | -0.05±0.07 | 0.08±0.06 |
| 6 mo | -1.34±1.01 | -1.42±0.98 | -0.13±0.07 | -1.29±0.96 | -0.03±0.06 | 0.09±0.06 |
| 9 mo | -1.58±0.98 | -1.67±1.00 | -0.13±0.06* | -1.52±0.97 | -0.03±0.07 | 0.10±0.07 |
| 12 mo | -1.74±0.96 | -1.89±0.98 | -0.19±0.07** | -1.74±0.97 | -0.06±0.06 | 0.13±0.06* |
| 18 mo | -2.07±0.91 | -2.21±0.98 | -0.18±0.07** | -2.12±0.95 | -0.08±0.06 | 0.09±0.06 |
| 24 mo | -2.04±0.89 | -2.24±0.96 | -0.23±0.07** | -2.10±0.91 | -0.10±0.07 | 0.12±0.06* |
| Length-for-age difference |  |  |  |  |  |  |
| 1 mo | -1.94±1.96 | -2.20±2.11 | -0.33±0.16* | -2.06±2.01 | -0.26±0.13* | 0.08±0.15 |
| 4 mo | -2.42±2.31 | -2.60±2.14 | -0.26±0.16 | -2.38±2.20 | -0.11±0.14 | 0.15±0.14 |
| 6 mo | -2.95±2.21 | -3.12±2.15 | -0.28±0.15 | -2.85±2.12 | -0.08±0.14 | 0.19±0.13 |
| 9 mo | -3.66±2.25 | -3.88±2.33 | -0.32±0.15* | -3.56±2.26 | -0.10±0.16 | 0.22±0.16 |
| 12 mo | -4.30±2.35 | -4.67±2.40 | -0.47±0.15** | -4.32±2.41 | -0.16±0.15 | 0.31±0.16 |
| 18 mo | -5.79±2.50 | -6.20±2.72 | -0.49±0.17** | -5.95±2.64 | -0.23±0.18 | 0.26±0.17 |
| 24 mo | -6.49±2.77 | -7.11±3.02 | -0.70±0.20** | -6.65±2.86 | -0.28±0.20 | 0.42±0.18* |

^1^ Values are % or mean ± SD. ^2^ Values are coefficient ± SEM from linear mixed model, test of simple effects comparing FFR+LNS and FFR+MNP to FFR+CSB and FFR+LNS to FFR+MNP. Models used imputed values for growth measurements taken > 1 mo after an age time point. **P* < 0.05, ** *P* < 0.01. CSB, corn-soy blend; FFR, full family ration; LNS, lipid-based nutrient supplement; MNP, micronutrient powder; NFR, no family ration; RFR, reduced family ration.
